# Correspondence

**Published:** 1897-03

**Authors:** 


### CORRESPONDENCE.

EDITORS JOURNAL OF COMPARATIVE MEDICINE, ETC.

DEAR SIRS: The *National Stockman and Farmer*, an agricultural paper published in Pittsburg, Buffalo, and Chicago, is a journal of large circulation and influence, and most of its articles are written by experienced, competent men, whose opinions can safely be regarded as sound. But for some unknown reason it has placed the management of its veterinary department in the hands of a man who has no standing in the veterinary profession and whose views display not only much ignorance, but such wilful intention to mislead that attention should be called to the fact.

In the issue of January 7th there is an article that purports to be a reply to a number of questions that have been submitted to

" V. S.," and all of them relating to the subject of bovine tuberculosis, and especially the New York situation in relation to this question. We do not desire to interfere in local issues, such as the questions that bear on the work of the Tuberculosis Commission of the New York State Board of Health—the points brought up in relation to that matter can be ignored—but certain general statements and announced facts regarding the general subject of tuberculosis of cattle are so absolutely false as to require authoritative contradiction.

Every new question must of necessity receive general discussion and much opposition before the true facts are generally understood, and this seems to apply with greater force to public-health questions than those of any class, and especially when the interests of public health and property rights conflict. In the discussion of tuberculosis of cattle that has gone on so actively in the agricultural press and at meetings of farmers during the last few years the conservative members of our profession who have been called upon to make plain statements of clearly established facts bearing on this subject have met with extreme and violent opposition from those who feared that a needless scare was being aroused for the benefit of place-hunters; but in every case where tuberculosis has appeared in the herd of a farmer and he has made a careful study of the demonstrated facts in relation to the disease, has considered its cause, its dangers to other cattle and the health of the consumers of dairy-products, the means that have been employed to combat it, and the value of which have been proven by numerous practical tests, conducted on a large scale, but one conclusion has, so far as the large observations of the writer goes, been reached. The fact that stock-owners *who have had practical experience with this disease* have so generally reached the same conclusions regarding it, should have great weight with those who have not had an opportunity to make a personal study of it, and who are inclined to follow misleading, malicious writings and opinions, such as those under consideration.

It is stated in this writing, for instance, that a vigorous quarantine practised by the farmer himself will do more toward heading off the diseases of a contagious nature than all the health boards in the whole State. It is quite true that unless farmers are careful and wish to have tuberculosis exterminated among their cattle it is more difficult to effect permanent good results than when the owner of the cattle co-operates with the authorities. But, nevertheless, the inefficiency of the method advised, which consists in removing from the herd a cow that has well-marked tuberculosis and has been coughing up and distributing the germs of consumption for a long time, is so evident that contradiction is entirely uncalled for.

Reference is made to "tubercular disease" of other animals, "from the house-cat up to the family horse," evidently for the purpose of creating the impression that tuberculosis is so common among animals other than cattle that the restriction of the disease in herds would amount to but little. It might be interesting to "V. S." to know that only six cases of tuberculosis in horses have been recorded in the United States, and that no cases of tuberculosis in cats in the United States are known, with the exception of animals inoculated artificially. The statement is also made that experiments have shown that under proper sanitary conditions animals may mingle with those that are diseased and not contract the disease. As a general statement this is false. It is undoubtedly true that some cattle may mingle with tuberculous associates and not contract the disease. It is also true that some people may mingle with cholera, smallpox, and yellow-fever patients and not contract their disease, but this does not in the least vitiate the strength of the very numerous observations that have been made and show that tuberculosis is *frequently* contracted by well-fed animals from their associates when the sanitary conditions are excellent. "V. S.'s" statement in reference to the danger of the transmission of tuberculosis from cattle to people through milk is exceedingly weak, but nevertheless it is so plausible as to create the impression in the mind of the uninformed reader that his contention is fully proven. He states that there is less tuberculosis among people now than formerly, notwithstanding the fact that milk is much more freely used. When attention is called to the advances that have been made in the knowledge of tuberculosis during the last few years, and the extreme measures that are taken by physicians and boards of health to restrict its spread by way of education and advice to consumptives and their families, it is strange that the disease is not less prevalent than it is.

If "V. S." could show that the general sanitary conditions in houses are no better now than twenty years ago, and that no more precautions are taken by consumptives to destroy their infectious discharges, and by their families to avoid infection, his argument would indeed be a powerful one in support of his ridiculous opinion. When it is known and has been proven by frequent experiments that tuberculosis can be carried from a tuberculous cow to calves, swine, and other experimental animals (even to such a comparatively immune animal as the horse, according to the observations of Bang), by the use of milk, and when it is known that a large percentage of children die of intestinal tuberculosis, which has anatomically about the same characteristics as are found in the experimental animals fed upon infectious milk, and when all sources of infection for the children are excluded as thoroughly as possible and nothing but milk from a tuberculous cow remains, a fair-minded man cannot doubt that many children are infected from this source. The statement that there is less tuberculosis among cattle now than formerly is absolutely false and contrary to all published opinions that are based on actual observation, and indisputable figures in many cases show that the disease is progressing steadily, excepting where active measures are used to restrict its progress. This is true of every portion of the United States and of European countries. The statement that all human and animal life would long since have been banished from the earth if tuberculosis were a highly contagious disease displays such ignorance that it is surprising that a reputable paper would publish it. Is not rinderpest a highly contagious disease, and do not contagious pleuro-pneumonia, foot-and-mouth disease, glanders, smallpox, cholera, yellow-fever, and diphtheria belong to this category, and do not animals and people still live?

The statement is made that there is no danger whatever of there being an outbreak of tuberculosis among the herds of cows or other animals in any State of the Union, and this in the face of the fact that tuberculosis prevails so extensively in some districts that from 10 to 25 per cent. of all the cows in dairies are involved, and in some herds nearly all of the cattle are tuberculous, while the general percentage of the tuberculous cattle throughout the dairy States cannot fall below 5 or 6, but still "There is no danger whatever that there will be an outbreak of tuberculosis among cattle in any State in the Union!"

Complimentary reference is made to the information that farmers have received on caring for their cattle, from the State Experiment Stations; but if "V. S." will merely refer to the publications from the Experiment Stations in Maine, Vermont, Massachustetts, New York, Pennsylvania, Indiana, Virginia, Michigan, Iowa, Utah, Arkansas, Alabama, and others, he will find careful treatises upon tuberculosis and the means to be employed in preventing it, and, strange as it may seem to "V. S.," the measures recommended by these various Experiment Stations are those that are recommended and enforced by State authorities wherever active measures are in operation. It is true, as "V. S." says, that there is no class of men who are as much frightened in regard to any disease that may attack the herd as the farmer himself; as a natural result of this, and the fact that tuberculosis is a dangerous disease that prevails extensively, there is great necessity that it shall be restricted in every way possible by the aid of the State.

It is stated in this remarkable effusion that in most cities to which milk is shipped milk-inspection is made by the different companies that handle the milk from the herds from which it comes, so that there is practically no danger to be apprehended by the city consumer that he will not get wholesome milk. The reverse of this is true, and no statement could be further from the mark. The milk coming from inspected herds constitutes such an exceedingly small percentage of the total supply of every large city, with the exception of St. Paul, Minnesota, that it is scarcely to be considered. Some of the herds in southern New York and northern Pennsylvania that supply large quantities of milk for New York City and some for Philadelphia are subjected to a so-called inspection at long intervals that amounts to nothing at all, and there is no such thing as a general dairy inspection made by competent men in operation in any of the Eastern, Southern, or Western States, with the exception of Minnesota.

It is very easy to say that tuberculosis is discussed and that some scientific men take an interest in the solutions of the intricate questions involved for the purpose of providing positions for "a great surplus of mighty poor doctors, lawyers, and half-educated dudes, who by reason of having friends who have a political pull are seeking to have a place provided for them." It is as fair to say that any other work is discussed or carried on for the same purpose. Are manufacturing establishments or railroads conducted for the primary purpose of giving men employment, and do all kinds of private and public work come within this category? Veterinarians as a class are as honest as any other class.

It has been shown that tuberculosis is a widespread disease among cattle; that it is a contagious disease, is progressively spreading and involving more cattle; and that it can be conveyed to other animals by feeding them upon the products of tuberculous cattle; and it is so highly probable that people can contract tuberculosis in this way that the highest medical authorities reckon tuberculous milk among the immediate causes of tuberculosis that should be guarded against in every possible way. In the face of all these well-demonstrated and generally accepted facts, how can an agricultural paper that has the interest of the farmers at heart publish and apparently approve of such infantile twaddle as the article of "V. S." printed in the *National Stockman and Farmer*, p. 11, of the edition of January 7, 1897? A. B. C.

